# The Effect of Work Engagement and Perceived Organizational Support on Turnover Intention among Nurses: A Meta-Analysis Based on the Price–Mueller Model

**DOI:** 10.1155/2023/3356620

**Published:** 2023-02-27

**Authors:** Li-Li Zhu, Hui-Juan Wang, Ya-Fei Xu, Shu-Ting Ma, Yan-Yan Luo

**Affiliations:** ^1^School of Nursing, Xinxiang Medical University, Xinxiang, China; ^2^School of Nursing, Xinxiang University, Xinxiang, China

## Abstract

**Aim:**

To elaborate on the relationship between work engagement, perceived organizational support, and the turnover intention of nurses by analysing some potential moderators.

**Background:**

Nurses' turnover intention is negatively impacted by their level of work engagement and perceptions of organizational support. However, it is challenging to reach a consistent conclusion.

**Methods:**

Data were acquired from six electronic databases. Each study was evaluated using the quality assessment tool for cross-sectional studies of the Agency for Healthcare Research and Quality (AHRQ). STATA 15.0 was used to analyse the data, and a random effects model was used. The groups that included two or more studies were added to the moderator analysis.

**Results:**

A total of 40 study articles involving 23,451 participants were included. The turnover intention of nurses was inversely associated with work engagement (coefficient: −0.42) and perceived organizational support (coefficient: −0.32). A substantial moderating role was played by cultural background, economic status, working years, and investigation time (*P* < 0.05).

**Conclusion:**

Work engagement and organizational support significantly reduced turnover intention among nurses. Considering the acute shortage of nurses worldwide, nurses with lower wages, fewer working years, and lower levels of work engagement should be given more attention and support from their organizations. *Implications for Nursing Management.* The meta-analysis suggested that managers should give their employees a more organizational support and promote their work engagement to motivate nurses' retention intention and maintain a stable workforce with little employee turnover.

## 1. Introduction

In 2021, the International Council of Nurses (ICN) proposed a policy brief indicating the need for 10.6 million additional nurses by 2030. However, the COVID-19 pandemic exacerbated the global nursing shortage and increased nurses' professional challenges, such as occupational risk of infection, stress, and a severe workload [1]. Nurses are experiencing increasing burnout, depression, and dissatisfaction with their profession. Subsequently, work engagement and quality of care declined, increasing the risk of medical errors and lower patient satisfaction. Researchers have even discovered an increase in the number of nurses leaving the profession. The Swedish Nurses Association announced that 7% of the nursing workforce (5,700 nurses) considered resigning due to the increased pressure and workloads brought on by the pandemic [2]. In Egypt, a study revealed that over 95% of nurses intended to leave their present jobs in a COVID-19 triage hospital [3]. Perceived organizational support (POS) is defined as employees' perception of the extent to which their organizations value their contributions and care about their well-being [4]. Increasing POS might help promote nurses' work engagement and reduce their burnout and turnover intention.

**Figure 1:**
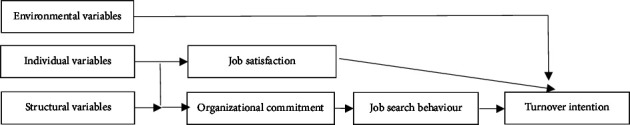
Price–Mueller model.

In analysing turnover factors, the Price–Mueller model is commonly used [5]. It contains four major domains: environmental, individual, structural, and intervening (Figure 1). Environmental variables represent constraints on intent to stay resulting from social conditions external to an organization. The individual variables mainly refer to training, job engagement, and emotions, with job engagement being the model's essential variable [6]. Structural variables include equity, job pressure, awards, and promotion opportunities. Award and promotion opportunities can directly predict turnover intention [7] and indirectly alter turnover intention through job satisfaction. The intervening factors mainly include job satisfaction, organizational commitment, job search behaviour, and turnover intention, which directly influence turnover behaviour. Among them, job satisfaction and organizational commitment are the essential influencing variables of turnover, and economists have noted job search behaviour.

Accordingly, our research is based on the Price–Mueller model, which primarily explores the impact of nurses' work engagement and perceived organizational support on turnover intentions from a positive psychological perspective.

## 2. Conceptual Relationships and Hypothesis Development

Turnover intention is a psychological and behavioural tendency that occurs when employees plan to leave their current organization or occupation. It is a crucial factor for predicting turnover behaviour directly [8].

### 2.1. Correlation between Turnover Intention and Work Engagement

Work engagement (WE) is a positive, fulfilling, work-related mental state with vigour, dedication, and absorption features [9]. Individuals who are deeply engaged in their work frequently have a strong sense of hope, meaning, pride, competence, progress, and a positive psychological experience. This higher level of job satisfaction, fulfilment, and recognition are necessary for employees, such as nurses, to remain in an organization. However, the relationship between work engagement and turnover intentions varies. An Australian study found a somewhat negative association between nurses' willingness to leave their jobs and their level of work engagement [10]. An Italian analysis revealed a moderately negative relationship between nurses' work engagement and turnover intention [11].

### 2.2. Correlation between Perceived Organizational Support and Turnover Intention

Perceived organizational support includes two types of rewards. Intrinsic rewards are found within the job role, whereas extrinsic rewards include pay, benefits, and advancement opportunities [12]. Research has shown that perceived organizational support positively affects nurses' job satisfaction, increases nurses' emotional attachment to the organization, and improves organizational commitment [13, 14]. It also fosters a sense of responsibility to contribute to the achievement of organizational advantages and goals, which lowers the likelihood of leaving. Research on organizational support and turnover intention has gradually increased in recent years. However, the effects of perceived organizational support on nurses' turnover intention were inconsistent. For example, one study found strong negative relationships between perceived organizational support and nurse turnover intention in China [15]. Perceived organizational support had a weak negative relation to hospital nurses' turnover intention in Egypt and Italy [16, 17]. In the UK, there was a moderately unfavourable association between nurses' intention to leave and the support they felt from the company [18].

### 2.3. Hypothesis Development and Research Questions

These abovementioned studies confirmed that perceived organizational support and work engagement were positive indicators of turnover intention. However, the strength of the relationship between these studies was inconsistent. The meta-analysis model was used to synthesize the effects of work engagement/perceived organizational support on nurse turnover intention.

  Question 1: What is the degree to which the intention to leave nursing is correlated with nurses' work engagement?
  Question 2: How strong is the link between nurses' perceived organizational support and their turnover intention?
  Question 3: Is there a relationship between turnover intention and work engagement and perceived organizational support, moderated by backgrounds, organizational characteristics, measurement instruments, and individual differences?

## 3. Methods

This study followed the Preferred Reporting Items for Systematic Reviews and Meta-Analyses (PRISMA) statement guidelines [19].

### 3.1. Literature Search

Relevant articles were systematically searched in scientific databases (PubMed, Embase, the Cochrane Library, Web of Science, Medline, and Scopus). The core search consisted of Medical Subject Headings (MeSH), Embase subject headings, and keywords. We used the subject headings and keywords “nurse” and “perceived organizational support” and “turnover intention;” “nurse” and “work engagement” and “turnover intention” to search the literature. A supporting information file presents detailed search strategies (see Supplementary Information S1). In addition, references to related articles were manually searched. The retrieval period was from their inception to July 5, 2022.

### 3.2. Inclusion and Exclusion Criteria

All eligible studies met the following criteria: (1) focused on nursing staff, (2) contained Pearson correlation coefficients for the relationship between work engagement and turnover intention or perceived organizational support and turnover intention, and (3) were articles published in English.

The following studies were excluded: (1) reviews, conference abstracts, incomplete data, and studies without full text, (2) studies repeatedly published literature, and (3) low-quality literature.

### 3.3. Study Selection

All search results were imported into EndNote X9 software. First, duplicates were eliminated according to titles and abstracts. Second, two researchers independently screened the full texts of the papers according to the inclusion and exclusion criteria.

### 3.4. Quality Assessment

To evaluate the quality of the included studies, two researchers independently used the quality assessment tool for cross-sectional studies recommended by the Agency for Healthcare Research and Quality (AHRQ) [20], a questionnaire containing 11 items. If an item was answered “YES,” it scored “1;” if the answer was “NO” or “UNCLEAR,” it scored “0”. Literature quality was evaluated as follows: high quality (8–11), medium quality (4–7), and low quality (0–3). Articles with quality scores below 4 were deleted.

### 3.5. Data Extraction and Statistical Methods

The following data were collected for each study: name of the first author, region (country), publication year, hospital type, number of participants, age, sex, education level, working years, evaluation tools, and the correlation coefficient between variables, as shown in Table 1. If there was missing or uncertain information, we tried to contact the original author to obtain it.

The analysis was conducted with STATA 15.0. For the correlation coefficient *r*: (1) the rs values in some studies were converted into *r* values; (2) the Fisher's *Z* values were converted by using the formula [21]; (3) the Fisher's *Z* values and the standard error SE were input into the STATA software, and the Summary Fisher's *Z* values were derived by using the inverse variance method, and finally, converted to Summary *r* values. We identified heterogeneity with the *Q* test and assessed it by *I*^2^ and *P* values. The *I*^2^ statistic represents the percentage of total variability between studies caused by heterogeneity. *I*^2^ values of 75%, 50%, and 25% correspond to high, medium, and low levels of heterogeneity, respectively. When the heterogeneity was greater than 50%, the random effects model was used. Publication bias was assessed by observing the symmetry of the funnel plot and Egger's test. *P* < 0.05 was considered to be statistically significant.

All authors worked together to discuss and analyse information from the included literature to identify the moderator. Individual differences, organizational characteristics, backgrounds, and measurement instruments are some possible moderators for the surveyed relationships. Finally, we combined the previous studies and the Price–Mueller model to summarize eight variables: culture, economy, survey time, hospital type, years of work, age, education level, and measurement instrument.

## 4. Results

### 4.1. Search Result

**Figure 2:**
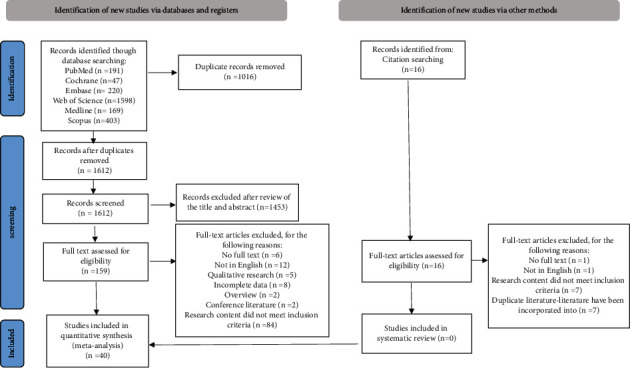
PRISMA flow diagram of the study selection process.

The PRISMA flow chart (Figure 2) describes the literature search and study selection process. A total of 2644 articles were retrieved from six databases and other sources. A total of 1628 remained after removing duplicates, and 175 full text articles were reviewed. Finally, 40 relevant articles were identified in the meta-analysis.

### 4.2. Characteristics of Studies and Participants

These studies were conducted in 18 different countries across 5 continents, with a sample of 24,351 nurses working in hospitals, medical institutions or nursing homes. In 31 studies, the female sex was significantly predominant (59.1%∼100%), while it was not mentioned in 9 studies. The average age and the average working years were the detailed features of each study, as shown in Table 1.

### 4.3. Data and Publication Bias

#### 4.3.1. Correlations between Work Engagement and Turnover Intention

**Figure 3:**
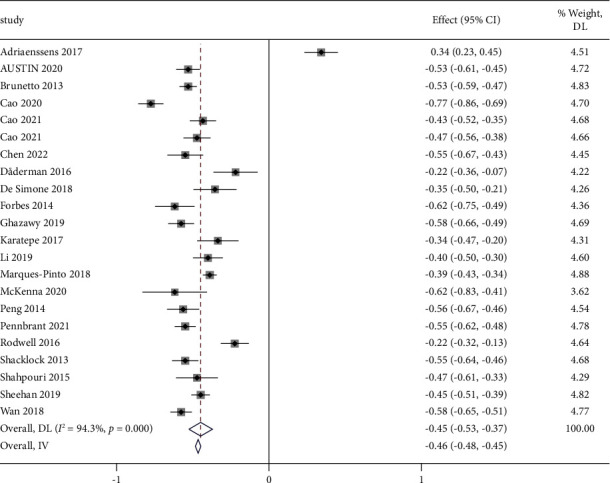
Forest plot of work engagement and turnover intention.

Of the 22 studies included in the review, 7 studies found a weakly correlated relationship between work engagement and turnover intention [11, 22–27]; 14 studies found a moderate correlation [15, 28–41]; and one study found a strong correlation [15]. We analysed the correlations between work engagement and turnover intention for 11,988 nurses. The *I*^2^ was 94.3%, indicating significant heterogeneity. Therefore, we used the random-effects model, as shown by the pooled Fisher's value of 0.45 (95% CI (−0.53, −0.37), *P* < 0.001) and the transformed *r* value of −0.42, 95% CI (−0.49, −0.35). The meta-analysis revealed that nurses' work engagement was moderately negatively correlated with their turnover intention (Figure 3).

**Figure 4:**
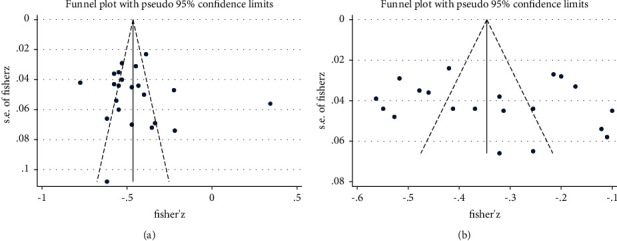
(a) Funnel plot of the work engagement and turnover intention. (b) Funnel plot of the perceived organizational support and turnover intention.

In addition, no publication bias was detected by Egger's test (*t* = 0.54, *P* = 0.595), with the funnel plot being substantially symmetrical in Figure 4(a).

#### 4.3.2. Correlations between Perceived Organizational Support and Turnover Intention

**Figure 5:**
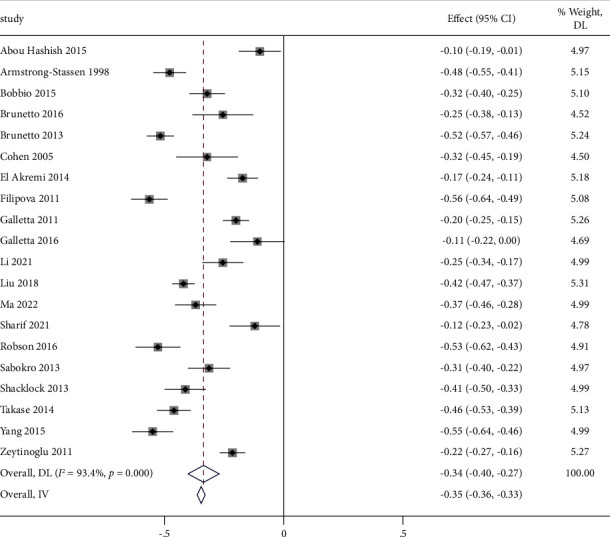
Forest plot of perceived organizational support and turnover intention.

Of the 20 studies included in the review, 5 studies found the relationship between perceived organizational support and turnover intention to be very weakly correlated [14, 16, 17, 42, 43], 9 studies found a weak correlation [13, 41, 44–50], and 6 studies found a moderate correlation [18, 41, 51–54]. We analysed correlations between perceived organizational support and turnover intention for 14,101 nurses. The *I*^2^ was 93.4%, indicating significant heterogeneity. Therefore, we used the random effects model, as shown by the pooled effect size Fisher's value of 0.34 (95% CI (−0.40, −0.27), *P* < 0.001) and the transformed *r* value of −0.32, 95% CI (−0.38, −0.26). The meta-analysis found that nurses' perceived organizational support was weakly negatively correlated with their turnover intention (Figure 5).

Using Egger's test, no publication bias was found (*t* = 0.48, *P*=0.638), and the funnel plot was symmetrical (Figure 4(b)).

### 4.4. Sensitivity Analysis

**Figure 6:**
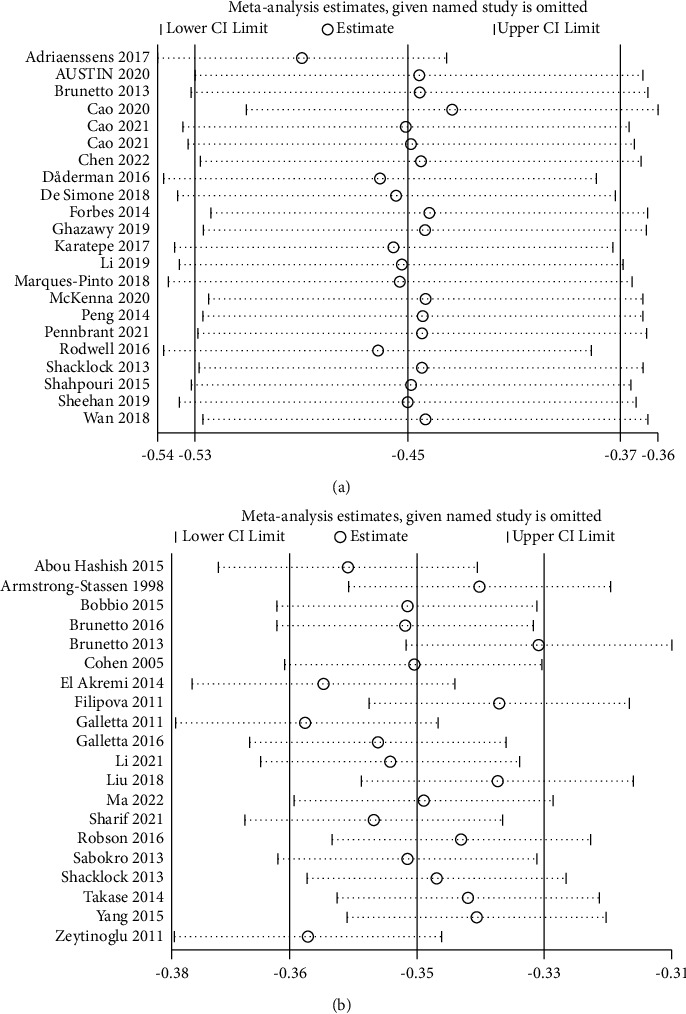
(a) Plot of sensitivity analysis of work engagement and turnover intention. (b) Plot of sensitivity analysis of perceived organizational support and turnover intention.

We performed sensitivity analysis by removing each study sequentially. These results indicated that in the overall meta-analysis, no single study significantly changed the pooled correlation coefficient (Figures 6(a) and 6(b)). The outcomes of the meta-analysis are statistically stable and reliable.

### 4.5. Moderator Analysis

The results of the moderator analysis are provided in Table 2. They revealed that regional culture, economic level, years of work, and survey time were significant moderators for work engagement and turnover intention (*P* < 0.05).

Table 3 shows the moderator analysis results of perceived organizational support and turnover intention. We found that regional culture and years of work were significant moderators for this relationship (*P* < 0.05).

## 5. Discussion

Over the last two decades, research concerning work engagement and perceived organizational support among nurses has increased. Work engagement and perceived organizational support have become crucial positive indicators of turnover intention. However, there is no meta-analysis based on theoretical models that research work engagement, organizational support, and contextual factors impacting turnover intention. This study aims to fill this gap. At the same time, this study verifies the Price–Muller model in the field of nursing.

Our meta-analysis indicates that the effect size of nurses' work engagement and turnover intention is moderate. Individual variables have a predictive effect on the turnover intention of nurses. Work engagement has a more significant impact on turnover intention among nurses than employees in other occupations. Zhang [55] found that the effect size of work engagement on turnover intention is weak among rural doctors in China. Work engagement motivates individuals to continue working. High work engagement implies a high level of energy and resilience [56]. Nurses work in a high-paced and demanding environment, and they must provide more technical and time-sensitive care to sicker patients. With the increasing demand for high-quality healthcare services, hospitals have emphasized the provision of healthcare that centres on patients' needs. Nurses must control their emotional expressions to match patients' experiences. Thus, nurses utilize behaviour and emotional labour to meet organizational goals through daily interactions with patients. Nurses need to show empathetic, sensitive, friendly, and caring emotional behaviour when interacting with patients and their families [57]. Nurses with low work engagement may perform less emotional labour, thereby consuming additional personal resources with negative effects, such as job burnout, emotional disorders, and exhaustion. In contrast, research shows that individuals with high work engagement actively change themselves to meet job demands [58]. Therefore, nurses with high work engagement do not need much cognitive processing or self-regulation. They can naturally express emotions to meet the goals of the organization, which reduces personal resource consumption and turnover intention.

Social exchange theory proposes that employees will exhibit beneficial behaviour towards the organization and reduce turnover when they receive affirmation and support from their organization [14]. According to the results of this study, the effect size of the relationship between perceived organizational support and turnover intention among nurses is negative. Structural variables have a predictive effect on the turnover intention of nurses. However, this relationship has a minor impact on other medical professions [59]. Nurses are primarily female. Due to the influence of traditional culture, women are the majority of family caregivers globally [60]. It is not easy to maintain work-family balance while working nursing shifts, doing housework, and performing childcare duties. Nurses need more support to promote the intention to maintain their jobs. Therefore, a high level of organizational support helps nurses reduce physical and mental stress, increase job satisfaction, and decrease turnover intention.

Our study shows that cultural background significantly moderates nurses' work engagement/organizational support and turnover intention. This difference may derive from the fact that employees define themselves and understand the rules in different cultures. For instance, in the East Asian cultural context, organizational support not only meets the need for employees to be valued and respected but also reinforces a sense of self-identity. Therefore, nurses' perceived organizational support is more sensitive to turnover intention.

Currently, global economic development is unbalanced. In developing countries, nurses' pay packages and working environments may not meet expectations [61]. Work engagement plays a vital role in the career planning of nurses, which is a motivation to continue working. Our study found that developing countries had a more significant effect on nurses' work engagement and turnover intention than developed countries. Therefore, the turnover intention of nurses was reduced. We should pay more attention to nurses' work engagement in developing countries. In addition, nurses who had worked for more than ten years had a lower sensitivity of work engagement/organizational support and turnover intention. It is possible that employees working for a long time had a strong emotional connection to the organization and a high level of organizational identification and loyalty. They, therefore, have a lower turnover intention.

It is worth noting that since 2020, nurses' work engagement has been more sensitive to turnover intention, probably due to the pandemic. Clinical nurses face negative stress reactions such as fear, anxiety, and burnout during long-term work. They need more motivating and positive emotions to continue working [62]. In addition, according to the moderator analysis results, survey time did not moderate the relationship between perceived organizational support and turnover intention. However, research shows that perceived organizational support can help reduce nurses' burnout during the COVID-19 pandemic [63] while promoting nurses' retention intention. Accordingly, during the COVID-19 pandemic, we should particularly focus on nurses' job engagement and perceived organizational support. Work together at individual and organizational levels to improve nurses' job satisfaction and reduce turnover intention.

### 5.1. Limitations and Future Research

Despite its comprehensive nature, this study also had limitations. First, the majority of the study samples generally consisted of female nurses; further research should involve more male nurses to enhance the universality of the results. It is worth noting that female nurses' marriage status was associated with turnover, but the information provided in this review is not suitable for moderating analysis. Second, we only included major databases in the literature search, and database search bias may exist. Finally, most of our studies were cross-sectional, and a few were longitudinal. Future researchers can summarize meaningful information from qualitative research.

## 6. Conclusion

This study revealed that work engagement and organizational support significantly reduced turnover intentions. This study allowed middle and senior nursing managers to gain more insight into the role of nurses' work engagement and organizational support. For example, nursing managers have always focused on patient safety issues worldwide, such as patient falls or medication errors. Adverse events not only cause physical and psychological damage to patients but also impose a great psychological burden on clinical nurses. At the same time, adverse events frequently cause turnover intention. Work engagement and perceived organizational support can reduce the rate of adverse events through both personal and organizational aspects and increase the job satisfaction of patients and nurses. High work engagement is closely related to medical quality results through a positive and highly dedicated working state. Organizational support can reduce nursing adverse events by improving the hospital safety management system. Meanwhile, when nurses experience adverse events as secondary victims, effective organizational support can alleviate nurses' anxiety, sleep disorders, and career burnout. Moreover, nurses' cultural background, economic level, working years, and investigation time played a significant moderating role in the surveyed relationships.

## 7. Implications for Nursing Management

Nursing shortages and high turnover rates have been the focus of nursing managers and an obstacle to addressing global public health challenges. In this regard, we have the following suggestions for middle and senior managers. First, hospital managers should change their thinking, attaching importance to the nursing role and enhancing nurses' positions to reduce the loss of talent. For instance, they can support the growth of nursing disciplines with policies and resources so that nurses can improve their career identification and engagement. Second, nursing managers should enhance humanistic care to improve nurses' satisfaction and to promote retention. (1) Managers should use inclusive and supportive ways to communicate, which contribute to nurses' physical and mental health. We suggest that nursing managers use leader gratitude expressions to communicate with nurses. As positive emotional communication, the leader's gratitude expression helps nurses form a positive evaluation of the organization, increase work satisfaction, and reduce turnover intention. (2) We propose encouraging nurses to have autonomous and elastic working time. For example, managers should upgrade working shifts to increase nurses' flexibility. Nurses can make appointments according to their needs in online systems to reduce family-work conflict. (3) Managers should identify the nurses' primary needs and effectively inspire nurses. While maintaining fair pay, managers should broaden the career development path of nurses and support nurses so they may participate in organizational affairs and decision-making that will increase their engagement. Finally, future studies should also focus on path analysis to assess the causal relationship between these variables.

## Figures and Tables

**Table 1:** Characteristics of studies and participants.

	Study	Country	Culture	Economy	Type of facility	Sample size	Average age	Bachelor or above	Working years	Measure tool	Variable
1	Abou Hashish 2015	Egypt	Islamic	Developing country	Hospital	500	—	35.00		POS	POS
2	Adriaenssens 2017	Belgium	Western	Developed country	Hospital	318	45.70	97.50	19.30	UWES	WE
3	Armstrong-Stassen 1998	Canada, Jordan	—	Developed country	Medical institution	826	33.58		7.68	POS	POS
4	Austin 2020	Canada	Western	Developed country	Medical institution	635	29.20	—	2.22	UWES	WE
5	Bobbio 2015	Italy	Western	Developed country	Public hospital	711	42.06	—	12.99	POS	POS
6	Brunetto 2016	Australian	Western	Developed country	Public hospital	242	38.20	59.60		POS	POS
7	Brunetto 2013	Australian,	Western	Developed country	Private hospital	1228	—	—	—	POS, Non-UWES	POS, WE
		USA									
8	Cao 2020	China	East Asian	Developing country	Public hospital	569	23.91	31.46	2.19	UWES	WE
9	Cao 2021	China	East Asian	Developing country	Public hospital	528	29.10	49.20	—	UWES	WE
10	Cao 2021	China	East Asian	Developing country	Public hospital	496	29.26	50.20	—	UWES	WE
11	Chen 2022	China	East Asian	Developing country	Public hospital	278	—	—	—	UWES	WE
12	Cohen 2005	Israeli	Western	Developed country	Public hospital	233	33.17	—	11.77	Non-POS	POS
13	Dåderman 2016	Poland	Western	Developed country	Hospital	188	41.00	—	19.50	UWES	WE
14	De Simone 2018	Italy	Western	Developed country	Public hospital	194	43.50	31.90	16.50	UWES	WE
15	El Akremi 2014	Italy	Western C	Developed country	Hospital	945	37.10	—	14.00	POS	POS
16	Filipova 2011	USA	Western	Developed country	Nursing home	656	—	—	—	POS	POS
17	Forbes 2014	UK	Western	Developed country	Medical institution	231	25.00	—	1.50	UWES	WE
18	Galletta 2011	Italy	Western	Developed country	Public hospital	1240	—	37.13	14.16	POS	POS
19	Galletta 2016	Italy	Western	Developed country	Hospital	304	35.60		12.40	POS	POS
20	Ghazawy 2019	Egypt	Islamic	Developing country	Public hospital	535	31.60	27.60	—	UWES	WE
21	Li 2021	Taiwan, China	East Asian	Developing country	Public hospital	512	—	—	—	Non-POS	POS
22	Karatepe 2017	Cyprus	Western	Developed country	Public hospital	212	—	69.80	—	UWES	WE
23	Li 2019	China	East Asian	Developing country	Community hospital	410	33.92	45.61	—	UWES	WE
24	Liu 2018	China	East Asian	Developing country	Public hospital	1716	—	59.40	—	POS-SVS	POS
25	Ma 2022	China	East Asian	Developing country	Public hospital	522			—	POS	POS
26	Marques-Pinto2018	Portugal	Western	Developed country	Hospital	1927	34.50	98.00	11.30	UWES	WE
27	McKenna 2020	Ireland	Western	Developed country	Hospital	89	—	—	—	UWES	WE
28	Sharif 2021	Iran	Islamic	Developing country	Public hospital	341	—	—	—	Non-POS	POS
29	Peng 014	Taiwan, China	East Asian	Developing country	Public hospital	349	31.80	36.10	7.90	UWES	WE
30	Pennbrant 2021	Sweden	Western	Developed country	Medical institution	807	42.00	—	13.80	UWES	WE
31	Robson 2016	UK	Western	Developed country	Medical institution	433	43.00	—	—	POS	POS
32	Rodwell 2016	Australian	Western	Developed country	Medical institution	459	—	—	—	Non-UWES	WE
33	Sabokro 2013	Iran	Islamic	Developing country	Hospital	494	—	—	—	Non-POS	POS
34	Shacklock 2013	Australian	Western	Developed country	Community hospital	510	46.50		—	Non-UWES,	WE, POS
										POS	
35	Shahpouri 2015	Iran	East Asian	Developing country	Private hospital	208	—		—	UWES	WE
36	Sheehan 2019	Australian	Western	Developed country	Medical institution	1039	48.67		22.47	UWES	WE
37	Takase 2014	Japan	East Asian	Developed country	Hospital	766	32.20	34.65	9.62	Non-POS	POS
38	Wan 2018	China	East Asian	Developing country	Public hospital	778	36.35	57.60	—	UWES	WE
39	Yang 2015	China	East Asian	Developing country	Public hospital	526		47.00	—	Non-POS	POS
40	Zeytinoglu 2011	Canada	Western	Developed country	Hospital	1396	42.00		18.00	Non-POS	POS

**Table 2:** Moderator analysis results/work engagement-turnover intention.

Concepts	*K*	*N*	*Z*score	Upper CI	Lower CI	*Q*	*P*	Egger's
Moderator (culture)	22	11988	−0.45^*∗∗*^	−0.53	−0.37	7.37	0.025^*∗*^	0.595
Islamic culture	1		−0.58	−0.66	−0.49			
Western culture	13		−0.39	−0.50	−0.27			
East Asian culture	8		−0.53	−0.62	−0.44			
Moderator (economy)	22	11988	−0.45^*∗∗*^	−0.53	−0.37	4.82	0.028^*∗*^	0.595
Developed country	13		−0.39	−0.50	−0.27			
Developing country	9		−0.54	−0.61	−0.46			
Moderator (hospital type)	22	11988	−0.45^*∗∗*^	−0.53	−0.37	2.98	0.084	0.595
Public hospital	9		−0.52	−0.61	−0.44			
Another type	13		−0.40	−0.51	−0.29			
Moderator (average age)	16	9514	−0.45^*∗∗*^	−0.54	−0.35	3.64	0.162	0.660
21∼30	5		−0.56	−0.69	−0.44			
31∼40	6		−0.51	−0.59	−0.43			
41∼50	5		−0.25	−0.55	0.06			
Moderator (bachelor's degree)	11	6316	−0.41^*∗∗*^	−0.55	−0.27	2.61	0.106	0.656
Below 50%	6		−0.52	−0.65	−0.39			
Above 50%	5		−0.29	−0.54	−0.04			
Moderator (years of work)	9	5908	−0.40^*∗∗*^	−0.56	−0.23	7.81	0.005^*∗*^	0.648
Less than 10 years	3		−0.64	−0.80	−0.48			
10+ years	6		−0.27	−0.47	−0.07			
Moderator (measurement instrument)	21	11776	−0.45^*∗∗*^	−0.53	−0.37	0.03	0.855	0.674
UWES	18		−0.46	−0.55	−0.36			
Non-UWES	3		−0.44	−0.62	−0.25			
Moderator (survey time)	22	11988	−0.45^*∗∗*^	−0.53	−0.37	5.56	0.018^*∗*^	0.595
2010∼2019	15		−0.40	−0.50	−0.30			
After 2020	7		−0.56	−0.65	−0.47			

^*∗∗*^ *P* < 0.001; ^*∗*^*P* < 0.05.

**Table 3:** Moderator analysis results/perceived organizational support-turnover intention.

Concepts	*K*	*N*	*Z*score	Upper CI	Lower CI	*Q*	*P*	Egger's
Moderator (culture)	19	13275	−0.33^*∗∗*^	−0.40	−0.26	8.20	0.017^*∗*^	0.662
Islamic culture	3		−0.18	−0.32	−0.04			
Western culture	11		−0.32	−0.42	−0.23			
East Asian culture	5		−0.41	−0.49	−0.33			
Moderator (economy)	20	14101	−0.34^*∗∗*^	−0.40	−0.27	0.4	0.527	0.638
Developed country	13		−0.35	−0.44	−0.27			
Developing country	7		−0.31	−0.42	−0.19			
Moderator (hospital type)	20	14101	−0.34^*∗∗*^	−0.40	−0.27	0.33	0.565	0.638
Public hospital	9		−0.31	−0.40	−0.23			
Another type	11		−0.35	−0.45	−0.25			
Moderator (average age)	10	5856	−0.33^*∗∗*^	−0.42	−0.24	0.43	0.511	0.714
31∼40	6		−0.30	−0.44	−0.17			
41∼50	4		−0.37	−0.50	−0.23			
Moderator (bachelor's degree)	6	4406	−0.33^*∗∗*^	−0.46	−0.20	0.03	0.872	0.438
Below 50%	4		−0.33	−0.53	−0.13			
Above 50%	2		−0.35	−0.51	−0.19			
Moderator (years of work)	8	6421	−0.29^*∗∗*^	−0.38	−0.19	45.07	<0.001^*∗∗*^	0.742
Less than 10 years	2		−0.47	−0.52	−0.42			
10+ years	6		−0.22	−0.27	−0.17			
Moderator (measurement instrument)	20	14101	−0.34^*∗∗*^	−0.40	−0.27	0.53	0.467	0.638
POS	15		−0.35	−0.43	−0.27			
Non-POS	5		−0.30	−0.41	−0.18			
Moderator (survey time)	20	14101	−0.34^*∗∗*^	−0.40	−0.27	2.45	0.294	0.638
Before 2010	2		−0.41	−0.56	−0.26			
2010∼2019	15		−0.34	−0.42	−0.26			
After 2020	3		−0.25	−0.39	−0.12			

^*∗∗*^ *P* < 0.001; ^*∗*^*P* < 0.05.

## Data Availability

The author does not wish to share the data.

## Supplementary Materials

Supplementary Materials Supplementary information S1 (search strategies).
