# Supplementary material for: The Effect of Work Engagement and Perceived Organizational Support on Turnover Intention among Nurses: A Meta-Analysis Based on the Price–Mueller Model
Source: J Nurs Manag. 2023 Feb 27;2023:3356620. doi: 10.1155/2023/3356620 (PMC11918525; doi:10.1155/2023/3356620)
Supplement: Supplementary Materials — Supplementary information S1 (search strategies). [file 3356620.f1.docx]

**Supplementary file**

**Search terms in PubMed database as an example.**

(1) Nurses’ work engagement and turnover intention.

Search: (((( "Nurses"[Mesh] ) OR "Nursing"[Mesh]) OR ((((nurse[Title/Abstract])) OR (nursing[Title/Abstract])) OR (health care workers[Title/Abstract]))) AND (("Work Engagement"[Mesh]) OR (((((job engagement[Title/Abstract])) OR (employee engagement[Title/Abstract])) OR (work engagement[Title/Abstract])) OR (engagement[Title/Abstract])))) AND ((((((((turnover intention[Title/Abstract])) OR (intention to leave[Title/Abstract])) OR (intent to leave[Title/Abstract])) OR (intention to quit[Title/Abstract])) OR (intent to quit[Title/Abstract])) OR (quit intent[Title/Abstract])) OR ( turnover[Title/Abstract]))

(2) Nurses’ perceived organization support and turnover intention.

Search: (((( "Nurses"[Mesh] ) OR "Nursing"[Mesh]) OR ((((nurse[Title/Abstract])) OR (nursing[Title/Abstract])) OR (health care workers[Title/Abstract]))) AND (((((((Perceived organization support[Title/Abstract])) OR (perceived organizational support[Title/Abstract])) OR (perceived organizational supports[Title/Abstract])) OR ( Organizational support[Title/Abstract])) OR (sense of organizational support[Title/Abstract])) OR (Perceived Support[Title/Abstract]))) AND ((((((((turnover intention[Title/Abstract])) OR (intention to leave[Title/Abstract])) OR (intent to leave[Title/Abstract])) OR (intention to quit[Title/Abstract])) OR (intent to quit[Title/Abstract])) OR (quit intent[Title/Abstract])) OR ( turnover[Title/Abstract]))
